# Quality of life of patients with Hirschsprung disease after Duhamel and Soave pull-through procedures: A mixed-methods sequential explanatory cohort study

**DOI:** 10.1016/j.amsu.2020.05.043

**Published:** 2020-06-12

**Authors:** Michelle Raj Saysoo, Fatwa Sari Tetra Dewi, Gunadi

**Affiliations:** a Medicine Study Program, Faculty of Medicine, Public Health and Nursing, Universitas Gadjah Mada, Yogyakarta, 55281, Indonesia; b Department of Health Behavior, Environment and Social Medicine, Faculty of Medicine, Public Health and Nursing, Universitas Gadjah Mada, Yogyakarta, 55281, Indonesia; c Pediatric Surgery Division, Department of Surgery, Faculty of Medicine, Public Health and Nursing, Universitas Gadjah Mada/Dr. Sardjito Hospital, Yogyakarta, 55281, Indonesia

**Keywords:** Duhamel procedure, Hirschsprung's disease, HAQL questionnaire, Quality of life, Mixed-method sequential explanatory cohort study, Soave pull-through, HAQL, Hirschsprung's disease/anorectal malformation quality of life questionnaire, HSCR, Hirschsprung disease, QoL, quality of life

## Abstract

**Background:**

Quality of life (QoL) is one of the important outcomes for patients with Hirschsprung disease (HSCR) after pull-through that provides qualitative data concerning the long-term outcomes, however, it has not been well-studied. The HSCR/anorectal malformation quality of life questionnaire (HAQL) is considered valid and reliable to evaluate the QoL of HSCR patients.

**Material and methods:**

A mixed-method sequential explanatory cohort study was conducted to compare the QoL of HSCR patients after Duhamel and Soave pull-through at Dr. Sardjito Hospital between 2013 and 2018 using an Indonesian adaptation of the HAQL, followed by a qualitative study.

**Results:**

We ascertained eleven HSCR patients (Duhamel: five HAQL parents and one HAQL adolescent *vs.* Soave: four HAQL parents and one HAQL adult). For the quantitative study, the mean HAQL score was 2.50 and 2.79 for the Duhamel and Soave groups, respectively. For the qualitative study, interviewed patients' parents expressed how their child's life had improved after surgery. However, frequent bloating was a major complaint following Soave surgery, whereas hardened stools were a major problem after Duhamel procedure.

**Conclusion:**

Here, for the first time using a mixed-method sequential explanatory cohort design, we show that patients with HSCR after Soave tended to have a higher overall QoL score compared to the Duhamel group. Further multicenter study with a larger sample size is mandatory to give better understanding about QoL of HSCR patients following pull-through.

## Highlights

• Quality of life (QoL) is one of the important outcomes for patients with HSCR after surgery, however, it has not been well-studied.
• The advantage of QoL studies is providing qualitative data concerning the long-term outcomes of patients after a surgical procedure.
• For the first time using a mixed-method sequential explanatory cohort design, we show HSCR patients after Soave tended to have a higher overall QoL score compared to the Duhamel group.

## 1 Introduction

Hirschsprung disease (HSCR) is a complex genetic disorder defined as the absence of ganglion cells in the distal bowel extending proximally over a varying length of the intestines, resulting in functional obstruction in infants [1]. Its incidence is approximately 1 in 3250 to 5000 live births [[1], [2], [3]].

Surgical strategy for HSCR is to remove the aganglionic intestines and connect normal ganglionic intestines as distally as possible [4]. The most common procedures for HSCR are the Soave and Duhamel procedures [[5], [6], [7]].

Quality of life (QoL) is one of the important outcomes for patients with HSCR after surgery [8]. The HSCR/anorectal malformation quality of life questionnaire (HAQL) is considered valid and reliable to evaluate the QoL of HSCR patients after definitive surgery in several languages [[9], [10], [11], [12]]. We determined the QoL of HSCR patients after Duhamel and Soave procedures using an Indonesian adaptation of HAQL. An explanatory qualitative study was done afterwards to know how patients experience their condition within their daily life.

## 2 Methods

### 2.1 Patient subjects

HSCR was diagnosed according to the clinical manifestations, contrast enema, and histopathological findings [2,5]. According to the current standard protocol, we utilized hematoxyline and eosin and S100 immunohistochemistry for the histopathological diagnosis of HSCR [2,5].

This cohort used a mixed-methods sequential explanatory research that consisted of a quantitative study followed by a qualitative study for patients with HSCR who underwent Duhamel and Soave procedures at Dr. Sardjito Hospital, Indonesia, from 2013 to 2018. During a 5-year period, 48 medical records of HSCR patients who underwent Duhamel (n = 20) and Soave (n = 28) pull-through at our institution were collected. One, 19 and 17 patients were excluded from this study because the patients' age was 1 year old, the parents’ patients were unable to be contacted and they were not willing to join the study, respectively.

The research was approved by the Institutional Review Board of the Faculty of Medicine, Public Health and Nursing, Universitas Gadjah Mada/Dr. Sardjito Hospital (Ref: KE/FK/0791/EC/2018). Consecutive sampling was used to recruit participants for this study. After signing the informed consent form to participate, patients’ parents were contacted by the attending physician either via phone calls or direct interview at the hospital during their visit to the outpatient clinic.

The work has been reported in line with the STROCSS criteria [13] and registered at Research Registry with UIN of researchregistry5218.

### 2.2 Soave and Duhamel procedures

Soave and Duhamel procedures were performed as previously reported [[5], [6], [7]]. Soave surgery involves removing the mucosa and submucosa of the rectum and pulling the ganglionic bowel down into the aganglionic muscular cuff of the rectum, followed by primary anastomosis above the dentate line [5,7]. Duhamel technique consists of resecting the aganglionic colon and bringing down the ganglionic colon via the retrorectal space, followed by colorectal anastomosis and elimination of the septum between the rectum and the ganglionic colon [5,6]. Soave and Duhamel procedures were chosen according to attending physician preference in our hospital.

### 2.3 The HSCR/Anorectal malformation quality of life questionnaire (HAQL)

We used the HAQL because it is already considered valid and reliable for evaluation of QoL of patients with HSCR in various populations using different languages [[9], [10], [11], [12]].

In the original HAQL, the minimum age of a patient to participate is 6 years old [9]. We modified the minimum age of participants to be 3 years old for two reasons: 1) more patients could take part since there is a limited number of participants from our institution for this study, and 2) by the age of three, most children are already given toilet training by their parents [5]. According to a previous report, the HAQL is divided into the parents-reported questionnaire for children aged 3–11 years, adolescent reported questionnaire for ages 12–16 years and adult reported questionnaire for ages 17 years and above [9].

The HAQL for parents, adolescents and adults used in this study had a total of 36 items to be answered by patients. These items were divided into 8 dimensions which were: laxative diet, constipating diet, presence of diarrhea, fecal continence, urinary continence, social functioning, emotional functioning and body image and physical functioning [9,10]. A score from 0 to 3 was given in response for each item and a better QoL was indicated by a higher score. For each of the dimensions, the scores were computed as the sum of the item scores which was then divided by the number of items answered in that dimension [10]. The overall QoL score was determined by calculating the total mean of the eight dimension scores for each type of surgery.

### 2.4 Qualitative study

Patient's parents from each type of surgery based on their availability were invited to participate. All interviews were conducted via telephone in Indonesian language to allow them to express their experiences with fewer communication barriers. The duration of the interview with the parents of patients who underwent Soave procedure was about 15 min while for the patients who underwent Duhamel procedure it was 13 min. Prior to the interview, the participants were briefed that their experiences after the surgery and contribution to the research may help other patients who have not completely recovered after the surgery to improve their QoL. Participants were asked open ended questions exploring their experiences related to their answers from the HAQL. The conversations were recorded for validation of the content. The interview was then translated from Indonesian to English and transcribed by the first author to be analyzed and used in the results of this study. The second author read through the transcript to identify meaning units related to the surgeries. Repeated patterns of how patients experience their daily life were developed from those content comparisons, including how the patients experienced life after surgery and what were the major problems faced after the pull-through procedure. Representative samples of the parents' quotations were selected to demonstrate the difference in the participants' responses.

## 3 Results

### 3.1 Baseline characteristics

We ascertained eleven HSCR patients (Duhamel: five HAQL parents and one HAQL adolescent *vs.* Soave: four HAQL parents and one HAQL adults) (Table 1).

**Table 1:** Characteristics of HSCR subjects who underwent Duhamel and Soave pull-through procedures at Dr. Sardjito Hospital, Indonesia.

Characteristics	Duhamel (n)	Soave (n)
Age (years)		
⁃ 3–11 (HAQL parents)	5	4
⁃ 12–16 (HAQL adolescent)	1	0
⁃ >16 (HAQL adults)	0	1
Gender		
⁃ Male	4	5
⁃ Female	2	0
Duration of follow-up after surgery (years)		
⁃ <3		
⁃ 3-5	3	0
⁃ >5	2	3
	1	2

HAQL, Hirschsprung's disease/anorectal malformation quality of life questionnaire.

### 3.2 Quantitative study

Here, we report the QoL of patients with HSCR after pull-through using an Indonesian adaptation of the HAQL. We found that the Soave group tended to have a higher overall score of HAQL than the Duhamel group (Table 2).

**Table 2:** Comparison of QoL between Duhamel and Soave pull-through patients based on each dimension using HAQL.

HAQL Dimensions	HAQL Scoring		
	Duhamel (n = 6) (mean ± SD)	Soave (n = 5) (mean ± SD)	Δ
Laxative diet	2.08	2.60	0.52
Constipating diet	2.50	3.00	0.50
Presence of diarrhea	2.58	2.60	0.02
Faecal continence	2.06	2.83	0.77
Urinary continence	2.96	3.00	0.04
Social functioning	2.81	3.00	0.19
Emotional functioning and body image	2.69	2.83	0.14
Physical functioning	2.30	2.43	0.13
Overall	2.50 **±** 0.33	2.79 **±** 0.22	0.29

Δ, HAQL scoring of Soave – HAQL scoring of Duhamel; HAQL, Hirschsprung's disease/anorectal malformation quality of life questionnaire; QoL, quality of life; SD, standard deviation.

Analysis of individual dimensions showed that in some dimensions such as presence of diarrhea and urinary continence, the scores for Duhamel pull-through patients and Soave pull-through patients were similar (Table 2). This result showed that the patients from both surgeries experienced somewhat similar effects for items under both dimensions. There was a large difference in QoL score between the two procedures for dimensions such as fecal continence, constipating diet and laxative diet compared to the scores of other dimensions (Table 2). Moreover, for soiling frequency which comes under the fecal continence dimension and voluntary bowel movement which comes under the physical functioning dimension, the Soave procedure group also tended to have a better score compared to the Duhamel group (Table 2). However, due to the small sample size used in this study, the statistical significance in the differences between the scores of the two procedures cannot be accurately determined.

### 3.3 Qualitative study

Following the quantitative study, a qualitative study was conducted which addressed major questions based on the HAQL dimensions to further explain how patient's experience their new condition after surgery and how to cope some difficulties. To know the condition of both procedures, we selected one patient of each procedure. We decided to interview the parents of selected patients in order to generate rich information due to better communication ability. As a representative sample for the Soave procedure group, quotations from one interview of a parent of a 7-year-old-male patient were selected, while for the Duhamel procedure group, one parent of a 5-year-old-male patient was used to demonstrate the difference in content. Those patients had the duration of follow-up after surgery before the interview of 5 years and 1 month and 2 years and 4 months for Soave and Duhamel patients, respectively.

During the interviews, the parents of both patients expressed that they felt that their child's life had improved after the surgery. This was shared by most parents in the study but some differences were expressed. The major problems faced by the patient post Soave pull-through was their child remained thin despite eating in large quantities (good appetite) and had frequent bloating.

“ … Do you know how big the portion of KFC rice is? Yes, that is still little for him … although he is eating and drinking a lot, he has a problem in his large intestine. Surgery has been done a few times for him … Sometimes the stomach is good and sometimes there is bloating too … Sometimes, during bloating, his flatulence gives out a very strong stench … If he is bloated, he has an explosive bowel movement.” (Mother of male Soave patient, 7 years old).

The parent of the Soave patient also mentioned that her child experienced fecal incontinence post-surgery very rarely.

“Waking up there are spots that look like feces. Yeah, like that but just a little. Even so, every day, at least once every 2 months or 3 months …” (Mother of male Soave patient, 7 years old).

Additionally, the parent of the Soave patient shared that she relied on home remedies to deal with problems in her child's daily life post-surgery. According to the parent, orange juice consumption usually relieves the hardened stools in her child. Besides that, to overcome her child's bloating stomach, the parent encourages her child to drink warm water first thing in the morning and positioned her child in a frog-like position to allow air from stomach to be released.

These experiences were different from that of the selected representative patient post Duhamel pull-through who reported hardened stools and poor appetite as the major problem.

“About defecating, you can't predict it. Not every day, maybe morning or afternoon or evening … he defecates at least two days once and the feces is hard, he feels pain … He doesn't want the food that children normally eat. He only eats rice, he doesn't want bread, doesn't want fruit. He doesn't want juice either. If he does want to try those things, he just wants a little.” (Mother of male Duhamel patient, 5 years old).

On the topic of fecal incontinence, the parent of the Duhamel patient said that at times, her child had experienced passing of feces in his diapers at night.

“At night, I still give diapers. Sometimes, at night while sleeping, he has a bowel movement in the diapers.” (Mother of male Duhamel patient, 5 years old).

## 4 Discussion

One advantage of QoL studies is providing qualitative data concerning the long-term outcomes of patients after a surgical procedure [8]. There are important effects on personal, social and professional spheres due to reoccurring constipation and/or fecal incontinence which will be reflected later during the patients’ life [8]. QoL data involve subjective information consisting of several areas that should be assessed individually and according to the expectations of patients and their relatives [8].

Most studies compared the QoL of HSCR patients with healthy controls [[14], [15], [16], [17], [18], [19]], while other studies focused on evaluation of one procedure for HSCR patients [20,21]. In one study, patients with HSCR had a lower psychosocial QoL scores, but not physical and total QoL scores, compared with healthy control children [14]. This difference was because the children with HSCR had poorer functional outcomes [14]. Interestingly, HSCR children scored lower QoL scores than the healthy children, whereas the adult patients showed significantly higher scores than their healthy counterparts [15]. This finding might be due to the adult patients having better coping approaches to face their symptoms than the children with HSCR [15]. Moreover, while patients showed poor functional outcomes, they showed a comparable overall QoL scores with healthy subjects [16,21].

To the best of our knowledge, our study is the first report that compared the QoL of patients with HSCR between two transabdominal surgeries, particularly the Soave and Duhamel procedures. One recent study compared the QoL between Duhamel and transanal Soave procedures and focused on the occurrence of failed pull-through [16]. We used the HAQL adapted into the Indonesian language for evaluation of QoL of our patients. This form might be useful for other populations with other Asian languages as well, besides those already translated [[9], [10], [11], [12]]. Another novelty of our study is using a mixed-methods sequential explanatory design to evaluate the QoL of HSCR patients after surgery.

Our qualitative study found that the QoL of both groups of patients were affected in similar ways that were commonly expressed in the interviews. Interestingly, as frequently found in Asian countries, some patients rely on traditional home remedies to deal with problems in their daily lives post-surgery.

However, in addition to some parents' reticence in the interviews, our findings should be interpreted cautiously because of the small sample size. The main reason was because many patients' parents were not willing to participate in this study since they did not find their participation in this study beneficial as their children's QoL had already improved after surgery.

## 5 Conclusions

Here, for the first time using a mixed-method sequential explanatory cohort design, we show that patients with HSCR after Soave tended to have a higher overall QoL score compared to the Duhamel group. Further multicenter study with a larger sample size is mandatory to give better understanding on QoL of HSCR patients after pull-through.

## Funding

The study was funded by the Indonesian Ministry of Research and Technology/National Agency for Research and Innovation.

## Ethical Approval

This study has been approved by the Ethical Committee of Faculty of Medicine, Public Health and Nursing, Universitas Gadjah Mada/Dr. Sardjito Hospital (Ref: KE/FK/0791/EC/2018).

## Author contribution

Michelle Raj Saysoo, Fatwa Sari Tetra Dewi and Gunadi conceived the study. Michelle Raj Saysoo conducted the study and drafted the manuscript, while Fatwa Sari Tetra Dewi and Gunadi critically revised the manuscript for important intellectual content. Gunadi facilitated all project-related tasks. All authors read and approved the final manuscript.

## Registration of Research Studies

Name of the registry: Research Registry

Unique Identifying number or registration ID: researchregistry5218

Hyperlink to your specific registration (must be publicly accessible and will be checked): https://www.researchregistry.com/browse-the-registry#home/registrationdetails/5dc50e541e687b001587986a/

## Guarantor

Gunadi

## Consent

Written informed consent was obtained from the parents before joining the study. A copy of the written consent is available for review by the Editor-in-Chief of this journal on request.

## Declaration of competing interest

No potential conflict of interest relevant to this article was reported.
